# Editorial: Traumatic brain injury and neurodegeneration: bridging the gap

**DOI:** 10.3389/fnins.2023.1197376

**Published:** 2023-04-21

**Authors:** Fatima Nasrallah, Nyoman D. Kurniawan

**Affiliations:** ^1^Functional Neuroimaging and Traumatic Brain Injury Laboratory, The Queensland Brain Institute, The University of Queensland, St Lucia, QLD, Australia; ^2^Centre for Advanced Imaging, Australian Institute for Biotechnology and Nanotechnology, The University of Queensland, St Lucia, QLD, Australia

**Keywords:** brain injury, microRNA, concussion, stroke, veterans, neuromodulation, meta-analysis, blast injury

Traumatic Brain Injury (TBI) is a serious condition that affects millions of people worldwide. It can result from various causes, such as trauma, stroke, infection, or degeneration, and can have lasting effects on brain function and structure. Brain injury can also increase the risk of developing neurodegenerative diseases, such as Alzheimer's disease (AD). Therefore, there is a need to understand the mechanisms, biomarkers, diagnosis, treatment, and recovery of brain injury and its consequences. In this paper collection, we present five studies that explore different aspects of brain injury using various techniques and methods.

The topic starts off by Lusardi et al. investigating the changes in microRNA levels in cerebrospinal fluid (csf) following traumatic brain injury in veterans and how they may be related to the risk of developing AD. By measuring miRNA levels in csf from three groups of participants: community controls with no lifetime history of TBI, deployed Iraq/Afghanistan veterans with no lifetime history of TBI, and deployed Iraq/Afghanistan veterans with a history of repetitive blast mTBI, the authors showed that a history of TBI increased the odds of developing AD.

This is complemented by Vorn et al.'s work which investigated the biomarkers of axonal injury in military personnel who were exposed to repetitive blast-related training. Using an ultrasensitive immunoassay platform, they found that serum p-tau181 levels were elevated after blast exposure on days 2 and 7 postblast injury, while serum tau and neurofilament light chain levels were elevated only on day 7. They suggested that blast exposure affected serum biomarkers indicating axonal injury, which could have implications for cognitive impairment and neurodegeneration.

The implications of a brain injury and its underlying effects was introduced by Gupta et al. where they investigated how TBI in adolescent rats affected the function of two types of neurons in the hippocampal dentate gyrus, a brain region involved in memory and learning. The authors found that TBI caused different and time-dependent inhibitory inputs into granule cells and semilunar granule cells, two projection neurons that send signals to other brain regions. The findings suggested that these changes in inhibitory inputs could contribute to the delayed emergence of cognitive deficits and seizure susceptibility after brain injury, providing novel insights into how TBI affects the function of dentate gyrus neurons, which could have implications for memory impairment and epileptogenesis after brain injury.

Cerebrovascular pathology is a key element in both TBI and AD, and therefore, the work by Qu et al. examined the relationship between TBI and stroke. The authors conducted a systematic review and meta-analysis of cohort studies that compared the risk of stroke (hemorrhagic or ischemic) in patients with prior TBI and those without. They found that TBI was associated with a more than two-fold increase in the risk of stroke, and the risk was much higher for hemorrhagic stroke than for ischemic stroke. The authors suggested that TBI may cause cerebrovascular damage, inflammation, and coagulation disorders that predispose to stroke. They also highlighted the need for more high-quality studies to explore the causal mechanisms and preventive strategies for stroke after TBI.

The final paper in the topic by Zhang et al. taps into the potential treatment, where it explored the changes in electroencephalography (EEG) metrics during recovery of consciousness in patients with disorders of consciousness (DOC) who underwent neuromodulation with high-density transcranial direct current stimulation (HD-tDCS). They found that HD-tDCS stimulation improved CRS-R scores and EEG metrics in the responder's group, indicating enhanced functional integration and segregation in the brain networks. They suggested that neuromodulation of the posterior lobe could lead to an EEG response related to consciousness in DOC, and that the posterior cortex might be one of the key brain areas involved in the formation or maintenance of consciousness. The paper provides evidence that HD-tDCS stimulation could modulate EEG metrics and improve consciousness in patients with DOC, which could have implications for severe TBI patients.

Collectively, these papers provide a comprehensive overview of the current state of knowledge and research on brain injury and its implications for brain function and structure. They also identify potential diagnostic tools and therapeutic strategies for improving the outcomes of brain injury. Findings from these papers may help clinicians and researchers in developing better ways to prevent, detect, treat, and rehabilitate brain injury.

## Author contributions

FN and NK have reviewed the articles and contributed to writing the editorial. All authors contributed to the article and approved the submitted version.

